# Effects of Lactofermented Beetroot Juice Alone or with *N*-nitroso-*N*-methylurea on Selected Metabolic Parameters, Composition of the Microbiota Adhering to the Gut Epithelium and Antioxidant Status of Rats

**DOI:** 10.3390/nu7075260

**Published:** 2015-07-16

**Authors:** Elżbieta Klewicka, Zenon Zduńczyk, Jerzy Juśkiewicz, Robert Klewicki

**Affiliations:** 1 Institute of Fermentation Technology and Microbiology, Faculty of Biotechnology and Food Sciences, Lodz University of Technology, 171/173 Wolczanska St., 90-924 Lodz, Poland; 2 Division of Food Science, Institute of Animal Reproduction and Food Research PAS, 10 Tuwima St., 10-748 Olsztyn, Poland; E-Mails: z.zdunczyk@pan.olsztyn.pl (Z.Z); j.juskiewicz@pan.olsztyn.pl (J.J.); 3 Institute of Chemical Technology of Food, Faculty of Biotechnology and Food Sciences, Lodz University of Technology, 4/10 Stefanowskiego St. 90-924 Lodz, Poland; E-Mail: robert.klewicki@p.lodz.pl

**Keywords:** beetroot juice, lactic acid fermentation, *Lactobacillus*, antioxidant status

## Abstract

An objective of this work was to assess the biological activity of beetroot juice (Chrobry variety, *Beta vulgaris* L. ssp. *vulgaris*), which was lactofermented by probiotic bacteria *Lactobacillus brevis* 0944 and *Lactobacillus paracasei* 0920. The oxidative status of blood serum, kidneys, and liver of rats consuming the fermented beetroot juice were determined. The experimental rats were divided into four groups on diet type: Basal diet, basal diet supplemented with fermented beetroot juice, basal diet and *N*-nitroso-*N*-methylurea treatment, and basal diet supplemented with fermented beetroot juice and *N*-nitroso-*N*-methylurea treatment. Mutagen *N*-nitroso-*N*-methylurea, which was added to diet in order to induce aberrant oxidative and biochemical processes and disadvantageous changes in the count and metabolic activity of the gut epithelium microbiota. The nutritional *in vivo* study showed that supplementing the diet of the rats with the lactofermented beetroot juice reduced the level of ammonia by 17% in the group treated with *N*-nitroso-*N*-methylurea. Furthermore, the positive modulation of the gut microflora and its metabolic activity was observed in groups of rats fed with the diet supplemented with the fermented beetroot juice. A concomitant decrease in the β-glucuronidase activity was a consequence of the gut epithelium microbiota modulation. The antioxidant capacity of blood serum aqueous fraction was increased by about 69% in the group of rats treated *N*-nitroso-*N*-methylurea mixed with the fermented beetroot juice and *N*-nitroso-*N*-methylurea *versus* to the *N*-nitroso-*N*-methylurea treatment, whereas the antioxidant parameters of the blood serum lipid fraction, kidneys, and liver remained unchanged.

## 1. Introduction

Red beetroot is a vegetable, willingly consumed in Europe. Its roots can be eaten raw or used as a valuable raw material in food industry for production of dried of frozen preserves, drinking and concentrated juices, and natural pigments (betacyanins), which are used as food additives. Beetroots contain biologically active betalains: Betacyanins and betaxanthins. Betacyanins are red-violet pigments: betanin, isobetanin, neobetanin, betanidin, and isobetanidin, while betaxanthins (red pigments) are represented by vulgaxanthin I and vulgaxanthin II. Rich sources of betacyanins are such plants as beetroot (*Beta vulgaris* L. ssp. *vulgaris*), cactuses of genera *Hylocereus* and *Opuntia*, and many flowering plant species of the family *Amaranthaceae* [1,2]. Beetroots are harvested seasonally and their biologically active, nutritious components may be lost during the storage. Therefore, processing methods providing maintenance of biologically active components and even their enrichment have been sought-after. Controlled lactic acid fermentation is one such method. The controlled lactic acid fermentation reduces concentrations of betanin and isobetanin and causes appearance of their aglycons—betanidin and isobetanidin [3].

In this study we attempted to evaluate the biological activity of beetroot juice derived by the controlled lactic acid fermentation using probiotic bacteria *Lactobacillus brevis* 0944 and *Lactobacillus paracasei* 0920. The oxidative status of aqueous and lipid fractions of blood serum, kidneys and liver of rats fed the fermented beetroot juice was determined. Previous research of the Authors dealt with protective properties of a fermented beetroot juice against aberrant crypt foci as well as metabolic changes after introduction of 2-amino-1-methyl-6-phenyllimidazo[4,5-*b*] pyridine (PHIP) biogenic amine into a diet. In the case of biogenic amines only products of metabolism or degradation are toxic, in contrast to *N*-nitroso-*N*-methylurea (MNU) which does not need a metabolic activation (its action is immediate, which ensures quick and noticeable toxic effect). Thus, the novel element of the research is a description of changes in the ecosystem, as well as in the intestinal metabolism, which are induced by a factor not demanding a metabolic activation. 

## 2. Experimental Section

### 2.1. Plant Material

Beetroot juice (Chrobry variety, *Beta vulgaris* L. ssp. *vulgaris*) was used in this study. The juice was produced under laboratory conditions with the productivity of 0.8 L of juice from 1 kg of beetroots. The juice was pasteurized for 10 min at 80 °C [4].

### 2.2. Microorganisms and Lactic Fermentation

Lactic acid bacteria were derived from the Collection of Industrial Microorganisms of the Institute of Fermentation Technology and Microbiology LOCK 105, Lodz University of Technology. A two strain inoculum, consisting of equal volumes of *Lactobacillus brevis* 0944 and *Lactobacillus paracasei* 0920 suspensions was used in the process of beetroot juice fermentation. The procedures of beetroot juice preparations and fermentation were thoroughly described by Klewicka *et al.* [5].

### 2.3. Animals, Chemicals and Diets

Four groups of Wistar rats (8 males in each group) were fed with the basal diet developed by Reeves for 8 weeks [6]. The animal protocol used in this study was prepared in compliance with European guidelines for the care and use of laboratory animals and was approved by the Ethical Committee for Animal Experiments in the northern region of Poland (Olsztyn, Poland, permision No. 68/2012, 24-10-2012). Animal husbandry conditions were described in the publication [5]. The 1st control group (C) was given only the basal diet, the 2nd control group (CJ) was fed with the basal diet and 3 mL lactofermented beetroot juice (FBJ). The 3rd group (M) was fed with the basal diet and MNU (Sigma Aldrich, St. Louis, MO, USA). The 4th group (MJ) was fed with the basal diet supplemented with 3 mL FBJ and MNU each. The rats were given MNU (50 mg/kg) diluted in 1% citric acid (ph = 3) twice—on day 23 and 26. The animals were sacrificed by carbon dioxide asphyxiation 30 days after the first carcinogen dose.

### 2.4. Antioxidant Capacity of Blood Serum

Antioxidant capacity of blood serum resulting from the presence of hydrophilic and lipophilic antioxidants was determined with a photochemiluminescence detection method using a Photochem (Analytik Jena AG, Jena, Germany). In the photochemiluminescence assay, the generation of free radicals was partially eliminated by the reaction with antioxidants present in serum samples, and the remaining radicals were quantified by luminescence generation. Ascorbate and Trolox calibration curves were used in order to evaluate hydrophilic and lipophilic antioxidants, respectively, and the results were expressed as μmol ascorbate (AA) or Trolox (T) equivalent per mL serum.

The level of lipids peroxidation (TBARS) was determined according to Ohkawa *et al.* [7]. Samples (0.3 g) of selected tissues (liver and kidneys) were suspended in phosphate buffered saline (PBS) (pH 7.0) in a proportion of 1:10 and homogenized for 1 min. Then the homogenates were vortexed and their 0.5 mL aliquots were mixed with 2.5 mL aliquots of 10% trichloroacetic acid (TCA), incubated in a boiling water bath (at 100 °C) for 15 min, cooled down for 10 min, and centrifuged at 25,000× *g* for 15 min. Supernatant aliquots (1 mL) were mixed with 0.5 mL of 0.67% thiobarbituric acid (TBA), incubated in a boiling water bath for 15 min and cooled down for 10 min. Then their absorbance was measured at a wavelength of 532 nm. Results were expressed as malondialdehyde (MDA) concentration (µmol/100 g tissue). MDA concentration was calculated based on standard curve.

### 2.5. Enzymatic Analysis

Mucosa from the second quarter of the small intestine was collected by scraping with glass slides on an iced glass plate. After homogenization with four parts of cold physiological saline (v/w) and centrifugation for 10 min (10,000× *g*, 4 °C), the obtained supernatant was stored at −20 °C until analysis. The mucosal disaccharidase activity (sucrase, maltase, lactase) was assayed using a procedure adopted [8]. An aliquot of mucosal homogenate (0.1 mL) was incubated at 37 °C with 0.1 mL of substrate solution (0.056 M sucrose, maltose or lactose in 0.2 M phosphate buffer, pH 7.0). After 30 min incubation, 0.8 mL of cold distilled water was added and the enzymatic reaction was interrupted by the immersion of the test tube in boiling water for 2 min. The blank probe with the same composition was prepared and immersed in boiling water without prior incubation at 37 °C. The released glucose was determined using a glucose oxidase reagent (Alpha Diagnostics Ltd., Warsaw, Poland). The disaccharides activity was expressed as μmol of glucose liberated from the respective disaccharide per min per g of protein. The mucosal protein content was estimated using the Bradford method, with bovine serum albumin as a standard [9]. Activities of microbial enzymes: β-glucuronidase (EC 3.2.1.31), β-glucosidase (EC 3.2.1.21), α- and β-galactosidase (EC 3.2.1.22 and EC 3.2.1.23) were described by Klewicka *et al.* [4].

### 2.6. The Ammonia Content and pH of Intestinal Digesta

Fresh cecal digesta were used for quantification of ammonia, which was extracted and trapped in a solution of boric acid and then determined by the direct titration with sulfuric acid [10]. The pH of the intestinal digesta was measured using a microelectrode and pH/ION meter (model 301; Hanna Instruments, Vila do Conde, Portugal).

### 2.7. Sample Preparation and Analysis by Fluorescent in Situ Hybridization (FISH)

Immediately after sampling, distal colon sections of about 0.5 g were taken. In order to remove non-adhered bacteria, the samples were washed three times by pouring 20 mL of sterilized PBS (pH 7.2) (Sigma Aldrich, St. Louis, MO, USA). The procedures of the FISH method preparation and genus-specific probe were thoroughly described by Klewicka *et al.* [11].

### 2.8. Statistic Analysis

The results of cecum parameters and antioxidant status of serum, liver and kidney of rats are expressed as the mean ± the standard error of the mean (SEM). A two-way analysis of variance (ANOVA) was used to determine the effects of diet (basal or mutagenic, D) and fermented juice (J) and the interaction between these two factors (D × J). If the analysis revealed a significant interaction (*p* ≤ 0.05), the differences among the treatment groups were then determined with Duncan’s *post hoc* test at *p* ≤ 0.05. The results of microorganisms adhering to intestinal epithelium and enzymatic analysis are expressed as the mean SEM. One-way analysis of variance (ANOVA) and the Bonferroni *post hoc* test (*p* ≤ 0.05) were applied to find differences between groups. 

## 3. Results

### 3.1. Cecum Parameters

Basic parameters of the cecum of experimental animals that were determined in this study are presented in Table 1. The relative cecal mass was virtually the same in all the groups and varied from 0.209 to 0.302 g/100 g body weight (BW). The mass of digesta in the control groups C and CJ was 1.025 and 1.014 g/100 g BW, respectively. It was slightly larger than that of the animals treated with the mutagen MNU (for group M of 0.985mg/g and for group MJ of 0.937 mg/g), however, the difference was not statistically significant. Two-way ANOVA showed that the concentration of ammonia in the cecal digesta was significantly reduced by the juice application.

**Table 1:** Cecum parameters of rats were fed mutagen *N*-nitroso-*N*-methylurea (MNU) and fermented beetroot juice.

Index	C	CJ	M	MJ	SEM	Diet		Juice		D × J
						C	M	0	+	*P*
Tissue mass ^1^	2.09	2.88	2.90	3.02	0.004	2.89	2.96	2.93	2.95	0.682
Digesta mass ^1^	10.25	10.14	9.85	9.37	0.028	10.20	9.61	10.05	9.76	0.947
NH_3_^2^	0.260 ^a^	0.209 ^b^	0.272 ^a^	0.226 ^c^	0.694	0.234	0.249	0.266 ^a^	0.217 ^b^	<0.05
pH of digesta	6.77	6.94	6.70	6.76	0.029	6.82	6.74	6.73	6.85	0.263

^1^ mg·g^−1^ body weight (BW); ^2^ mg/g digesta; C—Basic diet; CJ—Basic diet and fermented red beetroot juice; M—Basic diet and mutagen *N*-nitroso-*N*-methylurea (MNU); MJ—Basic diet; mutagen MNU and fermented red beetroot juice; ^a,b,c^—*p* ≤ 0.05; SEM—Standard errors of the means (standard deviation for all rats divided by square root of rat number, *n* *=* 32).

### 3.2. Intestinal Enzymes Activity

Activities of selected disaccharidases operating in the small intestinal mucosa (sucrase, maltase and lactase) and cecal glycolytic enzymes synthesized by the intestinal microbiota (β-glucuronidase, β-glucosidase, and α- and β-galactosidases) were presented in Table 2. The rats treated with MNU were characterized by a significantly lower sucrose and maltase activities as compared to the control dietary groups. Irrespective of the MNU treatment, the dietary application of the juice caused a statistical tendency towards decreased maltase activity in the small intestinal mucosa.

**Table 2:** Enzyme activity contents of the small intestine and cecum of rats were fed mutagen MNU and fermented beetroot juice.

Enzyme	Dietary Group			
	C	CJ	M	MJ
**Small Intestine—Mucosal Disaccharide Activity ^1^**				
Sucrase	31.1 (0.388) ^a^	29.3 (0.318) ^ab^	26.8 (0.848) ^b^	26.3 (0.709) ^b^
Maltase	98.4 (1.131) ^a^	85.3 (0.742) ^a^	78.7 (1.343) ^b^	76,7 (1.631) ^b^
Lactase	3.9 (0.318)	4.2 (0.353)	3.9 (0.141)	3.4 (0.176)
**Cecum—Microbial Glycolytic Activity ^2^**				
β-glucuronidase	11.8 (0.671) ^b^	9.5 (0.318) ^b^	23.0 (0.742) ^a^	11.0 (0.919) ^b^
β-glucosidase	12.0 (0,636)	12.6 (0.707)	15.5 (0.671)	14.4 (0.636)
α-galactosidase	18.1 (0.318) ^a^	25.8 (0.884) ^a^	21.7 (0.990) ^a^	46.2 (1.519) ^b^
β-galactosidase	53.4 (2.227)	51.5 (1.025)	56.7 (1.343)	55.9 (1.025)

^1^ μmol·min^−1^·g^−1^ of protein; ^2^ μmol·h^−1^·g^−1^ of digesta; ^a,b^ statistic significance, *p* ≤ 0.05; The results are expressed as mean and standard errors of the mean (SEM); *n* = 8 animals per dietary group; MNU—*N*-nitoso-*N*-metylourea; C—Basic diet; CJ—Basic diet and fermented beetroot juice; M—Basic diet and mutagen MNU; MJ—Basic diet, mutagen MNU and fermented red beetroot juice.

Two-way ANOVA indicated that both the MNU treatment and the juice application caused a significant increase in the α-galactosidase activity, so the highest activity of this enzyme released into the cecal environment by the intestinal microbiota was observed in the MJ animals. The diet type × juice interaction was significant in the case of β-glucuronidase activity, as the juice application significantly reduced the activity of that enzyme in rats treated with MNU, and not in the control ones.

### 3.3. Microorganisms Adhering to Intestinal Epithelium

Identification of microorganisms adhered to the gut epithelium by FISH method revealed the following groups: *Lactobacillus-Enterococcus* (probe Lab158), *Clostridium cocciodies* (Erec 484), enterobacteria (Enter 1432), *Bacteroides-Prevotella* (Bac 303), and *Bifidobacterium* (Bif 164), and enabled their enumeration (Eub 338). The most stable of these groups were bifidobacteria that almost equally colonized the gut epithelium of all the groups of experimental animals (their counts varied from 5.44 to 5.86 log_10_CFU/g) (Table 3). The presence of the mutagen in the gastrointestinal tract of the animals did not affect the count of microorganisms adhered to the gut epithelium. The count of *Lactobacillus-Enterococcus* bacteria adhered to the intestinal epithelium in the control group of animals (C) equaled 7.02 log_10_CFU/g of wet intestinal tissue. In the rats fed with the basal diet and the lacto-fermented beetroot juice (CJ group), the count of *Lactobacillus-Enterococcus* bacteria was increased to 7.56 log_10_CFU/g. In the groups treated with the mutagen, (M) and (MJ), counts of *Lactobacillus-Enterococcus* bacteria, adhered to the gut epithelium, were reduced to 6.64 log_10_CFU/g and 6.63 log_10_CFU/g, respectively. Thus, the fermented beetroot juice stimulated the adherence of *Bacteroides-Prevotella* bacteria to the gut epithelium. Their counts were higher in the groups treated with the juice (CJ and MJ) than in the control groups C and M. In the CJ group, the number of *Bacteroides-Prevotella* bacteria adhered to the gut epithelium was 0.7 log_10_CFU/g higher than in the C group, while for the groups MJ and M the difference equaled 0.23 log_10_CFU/g. In the groups C and CJ, the level of adherence of enterobacteria to the intestinal epithelium was similar, 6.02 and 5.95 log_10_CFU/g, respectively. In the group of rats treated with MNU (M) the number of adhered enterobacteria was increased to 7.25 log_10_CFU/g, while in the MJ group it equaled 6.36 log_10_CFU/g. Changes in the count of *Clostridium cocciodies* were observed only in the CJ group (a decrease by 1.01 log_10_CFU/g compared to the control group C). The total counts of bacteria, determined using the probe Eub 338, were 7.47 and 7.19 log_10_CFU/g in the control groups C and CJ, respectively, while in the groups M and MJ they were lower, 6.71 and 6.76 log_10_CFU/g, respectively.

### 3.4. Antioxidant Status of Serum, Liver and Kidney of Rats

A diet type by juice interaction was observed (*p* = 0.027) for hydrophilic antioxidants level in the serum (Table 4). The MNU treatment caused a significant hydrophilic antioxidants decrease and the dietary application of fermented beetroot juice enhanced hydrophilic antioxidants level in the MJ rats to the values observed in the control untreated animals. The serum hydrophilic antioxidants in the C and CJ groups were the same. The experimental treatment of animals with MNU caused a significant decrease in the lipophilic antioxidants serum level. The dietary application of juice had no effect on serum lipophilic antioxidants, irrespective of the diet type. The relative mass of the kidneys tended to be increased (*p* = 0.078) in MNU treated rats.

**Table 3:** The microorganisms which are able to adhere to the intestinal epithelium of rats fed with mutagen MNU and fermented beetroot juice in log_10_CFU·g^−1^ (SEM).

Probe	Dietary Group			
	C	CJ	M	MJ
Lab 158	7.02 (0.053) ^a^	7.56 (0.038) ^b^	6.64 (0.063) ^c^	6.63 (0.042) ^c^
Bac 303	7.47 (0.042) ^a^	8.17 (0.226) ^b^	6.89 (0.265) ^a^	7.12 (0.038) ^a^
Eub 338	7.47 (0.099) ^a^	7.19 (0.109) ^a^	6.71 (0.137) ^b^	6,76 (0.201) ^b^
Erec 484	6.58 (0.155) ^a^	5.57 (0.180) ^b^	6.79 (0.109) ^a^	6.37 (0.099) ^a^
Enter 1432	6.02 (0.060) ^a^	5.95 (0.113) ^a^	7.25 (0.042) ^b^	6.36 (0.162) ^c^
Bif 164	5.44 (0.127) ^a^	5.73 (0.109) ^a^	5.67 (0.131) ^a^	5.86 (0.053) ^a^

log_10_CFU·g^−1^—Logarithmic units per gram of wet intestinal tissue; (SEM) standard errors of the mean; C—Basic diet; CJ—Basic diet and fermented beetroot juice; M—Basic diet and mutagen *N*-nitroso-*N*-methylurea (MNU); MJ—basic diet and mutagen MNU and fermented red beetroot juice; ^a,b,c^—Statistical differences between groups (*p* ≤ 0.05).

**Table 4:** Antioxidant status of serum, liver and kidney of rats fed with alkylating agents (mutagen) MNU and fermented beetroot juice.

Index	C	CJ	M	MJ	SEM	Diet		Juice		D × J
						C	M	0	+	*P*
Hydrophilic antioxidants ^1^	0.043^a^	0.043 ^a^	0.023 ^b^	0.039 ^a^	0.002	0.043 ^a^	0.031 ^b^	0.033 ^b^	0.041 ^a^	<0.05
Lipophilic antioxidants ^2^	0.061^a^	0.066 ^a^	0.048 ^b^	0.048^b^	0.002	0.063 ^a^	0.048 ^b^	0.054 ^b^	0.057 ^b^	<0.05
Liver ^3^	36.88	35.59	36.41	37.13	0.040	36.24	36.77	36.64	36.36	0.414
MDA ^4^	30.15	29.84	29.43	30.87	0.854	30.00	30.15	27.79	30.36	0.530
Kidneys 3	5.99	5.88	6.22	6.33	0.008	5.93	6.28	6.11	6.11	0.837
MDA 4	85.84	86.18	84.08	86.08	2.250	86.01	85.17	84.96	86.13	0.550

^1^ μmol Ascorbic Acid (AA) equivalent per mL serum; ^2^ μmol Trolox (T) equivalent per mL serum; ^3^ mg·g^−1^ body weight (BW); ^4^ nmol·g^−1^ tissue; MDA, malondialdehyde; C, Control—Basic diet; CJ—Basic diet and fermented red beetroot juice; M—Basic diet and mutagen *N*-nitroso-*N*-methylurea (MNU); MJ—Basic diet and mutagen MNU and fermented red beetroot juice; ^a,b,c^—*p* ≤ 0.05; SEM—Standard errors of the means (standard deviation for all rats divided by square root of rat number, *n* = 32).

## 4. Discussion

This study focused on the impact of the lactofermented beetroot juice consumption on enzymatic and microbiological parameters of intestines of experimental animals. Additionally, the antioxidant parameters of blood serum and organs involved in the detoxication of organisms (kidneys and liver) were determined. The harmful factor used in the study was the mutagen MNU, which does not need any metabolic activation to act as a mutagen. The fresh and lactofermented beetroot juices are characterized by the high anti-carcinogenic and anti-mutagenic potentials [5,12,13]. The dominating betacyanins of the lactofermented beetroot juice are betanidin and betanin, while the fresh juice does not contain betanidin and the dominating compound is betanin [3]. Betanidin is the betalain with the highest antiradical activity described, due to the connection of the aromatic resonance system present in the indoline substructure to betalamic acid and to the presence of two hydroxyl groups [14]. The present study revealed an increase in the antioxidant capacity of the aqueous fraction of blood serum in these groups of rats, which were administered the lactofermented beetroot juice. The action of betanin is known to be limited only to the lipid phase, while betanidin displays the antioxidant activity both in the hydrophilic and lipophilic fractions [15]. Biological activity and bioavailability of betalains depend on environmental factors inside the gastrointestinal tract of humans and other animals. The knowledge of absorption of betacyanins in human organism is scarce. It is known that betalains from fruits of cactuses are gradually degraded in the human alimentary tract. Their amounts are decreased by 24%–29% in the stomach, by 20%–26% in the small intestine, and by 20%–29% in the large intestine [16]. Only betanin was identified in their blood serum. The large intestine digesta (sampled from the distal part) of the experimental rats given the lactofermented beetroot juice contained 39 ± 12.8 µg/g cecal digesta of betacyanin (around 1.6% of the original dose). High-performance liquid chromatography (HPLC) analysis of betacyanins extracted from the cecal digesta revealed mainly betanidins and isobetanidins, and minor amounts of betanin, which may imply that betanin was either completely or partly absorbed, or was degraded by enzymes of the gut epithelium or enzymes synthesized by the intestinal microbiota [17].

The normalizing effect of lactofermented beetroot juice on the cecal ecosystem was indicated by the lowest ammonia concentration in the digesta. The control (C) and mutagen (M) treatments gave rise to the highest concentration of ammonia in the cecum. In the corresponding groups, treated with the lacto-fermented beetroot juice, a decrease in the concentration of ammonia, of 20% (CJ) and 17% (MJ) was noticed. A decrease in amount of ammonia is important because this compound is harmful to cells of the gut epithelium. High ammonia concentrations in the intestine may stimulate the development of such diseases as liver encefalopathy and tumorigenesis [18,19]. One of the promoters of aberrant processes in cells of gut epithelium (formation of tumors) is the disadvantageous system of intestinal microbiota and its metabolic activity. Previous investigations of Klewicka *et al.* [4] showed that consumption of the lactofermented beetroot juice, containing live bacteria of the genus *Lactobacillus*, caused modulation of intestinal biocenosis and reduced formation of aberrant krypt foci (ACF), induced by MNU. Changes in the biocenosis of cecal digesta mainly consisted of a decrease in the count of *Enterobacteriaceae* family bacteria [4]. Variation in counts of bacteria adhered to the gut epithelium was observed in this study. In the groups of rats treated with the lactofermented beetroot juice, the counts of *Bacteroides-Prevotella* bacteria were increased and stabilized while the counts of *Clostridium coccoides* and enterobacteria were decreased. The study of Wrzosek *et al.* [20] revealed that commensal bacteria can influence goblet cells and mucin composition in the gut, providing new information about the relation among mucus, bacteria and intestinal homeostasis. *Bacteroides thetaiotaomicron* enhances goblet cell differentiation leading to an increase of goblet cell number and mucin gene expression in the colon of gnotobiotic rats. The presence of *B. thetaiotaomicron* also affects the composition of mucin O-glycans, with relative decreases in sulfated and neutral oligosaccharides in favor of sialylated oligosaccharides. *B. thetaiotaomicron*, therefore, appears to provoke modifications in the secretory lineage compared to Germ Free (GF) rats, favoring mucus production and they put the hypothesis that this is possibly for its own benefit. In the CJ group an increase in the count of *Lactobacillus-Enterococcus* bacteria was observed (by 0.54 log_10_CFU·g^−1^). The presence of mutagen in the animal diet (MJ group) caused a decrease in counts of the above-mentioned bacteria by 0.93 log_10_CFU·g^−1^. This reduction may be a result of detoxifying abilities of *Lactobacillus* sp. It is known that lactic acid bacteria are able to adsorb some toxic substances (for example: *p*-cresol, heterocyclic aromatic amines and ochratoxin A) to their cell wall [21,22,23]. Thus, the drop in the count of *Lactobacillus-Enterococcus* bacteria may be an after-effect of interaction between mutagen and bacterial cells. As a result, there was a decrease in the ability of the bacterium to adhere to the intestine epithelium. This phenomenon should be considered as positive in view of possible detoxification of intestine environment performed by *Lactobacillus* bacterium.

A decrease in β-glucuronidase activity in groups of rats fed with the lacto-fermented beetroot juice is an advantageous and desirable phenomenon. Most efficient producers of this enzyme are bacteria of genera: *Bacteroides*, *Enterococcus*, *Clostridium*, and *Eubacterium*, as well as *E. coli* [24]. However, β-glucuronidase is also synthesized by cells of animal tissues. Human β-glucuronidase is mainly found in the lysosomes and microsomes of normal tissues, with plasma levels of enzyme quite low. Moreover, human β-glucuronidase is optimally active at a lower pH, around 5.5, while the optimum pH for activity of β-glucuronidase from *E. coli* is around 7.0–7.4 [25]. The pH of intestinal digesta of experimental rats varied from 6.70 to 6.94. The probiotic *Lactobacillus* bacteria from the lactofermented beetroot juice neither stimulated fermentation processes in the intestinal digesta nor decreased their pH. However, the adherence of bacteria exhibiting the high β-glucuronidase activity (enterobacteria and *Clostridium coccoides*) to the gut epithelium was reduced.

The lactofermented beetroot juice containing live bacteria of the genus *Lactobacillus* is a product combines the biological activity of betacyanins, involved in limitation of oxidative processes in the organism, and activity of live bacteria modulating the composition and metabolic activity of intestinal microbiota. Health benefits caused by consumption of the lactofermented beetroot juice are diverse and consist of reducing oxidation processes in the organism and advantageous modulation of intestinal ecosystem and its enzymatic activities.

## 5. Conclusions

The lactofermented beetroot juice containing live bacteria of the genus *Lactobacillus* may be considered of functional foods. This product combines the biological activity of betacyanins, involved in limitation of oxidative processes in the organism, and activity of live bacteria modulating the composition and metabolic activity of intestinal microbiota.
